# The Effect of COVID-19 on QTc Prolongation

**DOI:** 10.7759/cureus.29863

**Published:** 2022-10-03

**Authors:** Isaac Alsallamin, Ewelina Skomorochow, Rami Musallam, Ameed Bawwab, Afnan Alsallamin

**Affiliations:** 1 Internal Medicine, Northeast Ohio Medical University, Cleveland, USA; 2 Internal Medicine, St. Vincent Charity Medical Center, Cleveland, USA

**Keywords:** covid-19, ecg abnormalities, cardiac arrhythmia, intraoperative arrhythmia, heart failure, qtc prolongation

## Abstract

Background

The severe acute respiratory syndrome coronavirus 2 (SARS-CoV-2) uses angiotensin-converting enzyme-2 receptors on host cells to enter the cells. These receptors are expressed on heart muscle tissue and the tissues of other major organs, which supports the primary accepted theory for the direct cardiac cell injury of coronavirus disease 2019 (COVID-19) and the associated cardiorespiratory manifestations. The SARS-CoV-2 infection leads to unstable myocardial cell membranes due to hypoxia, myocarditis, myocardial ischemia, and abnormal host immune response. This is the main reason behind arrhythmia and electrocardiogram (ECG) changes during COVID-19. However, the specific effect on QTc has not been studied well. Therefore, this study aimed to examine the association between COVID-19 and QTc changes.

Methodology

We conducted an observational, retrospective review of hospital medical records of 320 adult participants diagnosed with COVID-19 at our facility. After applying the exclusion criteria, 130 participants were included and distributed into two groups. One group had long QTc, and one group had normal QTc. Data were collected and recorded using Microsoft Excel. We used SPSS Statistics for Windows, Version 20.0. (IBM Corp., Armonk, NY, USA) to analyze the data. Student’s t-tests were performed for independent groups. Quantitative data were summarized using mean and standard deviation. Statistical significance was taken as p < 0.05.

Results

A total of 63 (48.4%) participants met the criteria for long QTc, and 67 (51.5%) participants had normal QTc (p < 0.001). There was no statistically significant difference in mortality outcomes between long QTc and normal QTc (0.8% vs. 3.8%, respectively; p = 0.21).

Conclusions

This study aimed to examine the association between COVID-19 and QTc changes. Nearly half of the participants had an increased QTc with COVID-19, and QTc length was not associated with mortality outcomes. Our results indicate that COVID-19 is an independent risk factor for QTc prolongation on ECG. Identifying COVID-19 as an independent risk factor for QTc prolongation is a clinically significant finding, and physicians should consider this when treating cardiac patients and possible COVID-19-positive patients.

## Introduction

Since the initial report of coronavirus disease 2019 (COVID-19) in February 2020, the severe acute respiratory syndrome coronavirus 2 (SARS-CoV-2) has spread rapidly. The number of cases has risen exponentially globally, with over 100 million infections and over two million deaths worldwide. As of April 2022, the total confirmed cases in the United States alone were 80,208,810, with 982,576 deaths [1].

The causes of death and poor outcomes in COVID-19 are not only limited to pulmonary complications such as hypoxic respiratory failure, acute respiratory distress syndrome, pulmonary embolism, and multiple organ failure [2,3]. Cardiac complications are the main cause of death; the relevant cardiac complications include heart failure, myocarditis, and stress cardiomyopathy [4,5]. Some studies have also reported COVID-19-related cardiac arrhythmias such as tachycardia, atrial fibrillation, supraventricular tachycardia, ventricular tachycardia (VT)/ventricular fibrillation (Vfib), and QTc prolongation as related to fatal COVID-19 outcomes [6-9]. The risk of Vt/Vfib is directly proportional to the QTc duration, especially at prolongations of >500 ms [10]. Others have reported the effects of COVID-19 on the heart muscle, causing cardiac muscle injury and leaking biomarkers such as troponin I and B-type natriuretic peptide (BNP) [11-14]. Although studies have examined the relationship between COVID-19 and treatment effects on QTc prolongation [15,16], they have not examined the direct relationship between COVID-19 and QTc prolongation. Therefore, we conducted this study to evaluate the direct effects of COVID-19 on QTc duration and prognosis.

## Materials and methods

We conducted a retrospective hospital patient medical record-based observational study. A total of 320 patients aged 18 years and older who tested positive for SARS-CoV-2 infection via polymerase chain reaction testing at our facility were included in the study; all were inpatients. Patients were excluded if they were missing electrocardiogram (ECG) measurements, had a baseline abnormal ECG or prolonged QTc or pacing rhythm, were lost to follow-up, or were stable and discharged home from the emergency department for home isolation. After applying the exclusion criteria, 130 patients remained in the study.

We used QTc parameters following the American Heart Association criteria for prolonged QTc as >450 ms in men and >460 ms in women. Automated QTc values calculated by telemonitors and ECG machines were noted, as previous studies have shown acceptable sensitivity and specificity of this method to predict and calculate QTc [17-19]. All automated prolonged QTc values were confirmed with manual methods by measuring QTc in lead II or V5 on a standard 12-lead ECG using the Bazett formula: QTc = QT/√RR.

Data were collected and analyzed over a 14-month period between April 2021 and June 2022. The control group included patients admitted for COVID-19 infection with normal QTc on admission ECG. We analyzed patients’ demographic distribution (e.g., age, gender, and race), electrolyte abnormalities, the use of medications that may prolong QTc, comorbidities, hypertension (HTN), diabetes, end-stage renal disease (ESRD), heart failure, coronary artery disease (CAD), and structural lung disease. We also assessed hypokalemia, hypomagnesemia, and hypocalcemia. Data were distributed into the following two groups: one group for normal QTc (i.e., <450 ms for men, <460 ms for women), and one group for long QTc (>451 ms for men, >461 ms for women). The longest QTc recorded during the hospital course within 14 days from symptom onset was included in the second group. This was also compared with QTc values documented on the most recent ECG within four to sixteen weeks before SARS-CoV-2 infection and one to nine months after infection.

Statistical analysis

For categorical data, results are shown as counts and percentages (%), with preliminary comparisons between groups done with Fisher’s exact test. The data were analyzed using SPSS Statistics for Windows, Version 20.0. (IBM Corp., Armonk, NY, USA). Quantitative data were summarized using mean and standard deviation, the maximum, minimum, median (50th percentile), and the number of observations. The comparisons between the QTc groups were made using the Student’s t-test for independent groups as the empirical distributions of that data did not indicate any non-normality.

P-values less than 0.05 were considered statistically significant. There were multiple testings of outcome data arising from individual patients. The p-values for univariate statistical tests were not corrected for multiple testing because those tests were exploratory. The subsequent multivariable logistic regression analysis was considered the main definitive result because it determined those variables as independently associated with the QTc group after adjusting for the contributions of the other variables.

## Results

Of the 320 participants, 130 met the study’s inclusion criteria. A total of 63 (48.4%) participants met the criteria for long QTc, and 67 (51.5%) participants had normal QTc (p < 0.001). Statistically significant QTc prolongation was noted during COVID-19 (p = 0.001). A significantly greater percentage of cases had persistent QTc prolongation after one to nine months of discharge (18.4%) compared to patients with normal QTc (13%; p = 0.04). Participants with long QTc (48.5%) were inpatients and had more comorbidities than normal QTc patients (45.4%; p = 0.006) (Table 1).

**Table 1:** Patient characteristics, lab results, and associated factors and comparison of patients with and without QTc prolongation. *: p < 0.05; **: p < 0.01. HTN: hypertension; HF: heart failure; CAD: coronary artery disease; ESRD: end-stage renal disease; SD: standard deviation; HR: heart rate; bpm: beats per minute; COPD: chronic obstructive pulmonary disease

Baseline characteristics	Total (N = 130)	Long QTc (n = 63)	Normal QTc (n = 67)	P-value
Mean age (years)	62.6	64.2	61.2	0.29
Gender				
Male, n (%)	69 (53.5%)	36 (27.9%)	33 (25.6%)	0.48
Female, n (%)	60 (46.5%)	27 (20.9%)	33 (25.0%)	
Race				
White, n (%)	31 (23.8%)	16 (12.3%)	15 (11.5%)	0.54
Black, n (%)	89 (68.4%)	40 (30.7%)	49 (37.6%)	
Other, n (%)	10 (7.6%)	6 (4%)	4 (3%)	
Comorbidities				
HTN, n (%)	87 (66.9%)	45 (34.6%)	42 (32.3%)	0.35
Diabetes, n (%)	49 (37.7%)	32 (24.6%)	17 (13.1%)	0.004**
HF, n (%)	19 (14.6%)	15 (11.5%)	4 (3.1%)	0.005**
CAD, n (%)	20 (15.4%)	11 (8.5%)	9 (6.9%)	0.63
ESRD, n (%)	14 (10.8%)	10 (7.7%)	4 (3.1%)	0.091
Mean heart rate (±SD)	92.3 (16.5)	95.48 (19.1)	89.33 (13.1)	0.036*
Tachycardia (HR >90 bpm)	72 (55%)	40 (30.8%)	32 (24.6%)	0.080
COPD, asthma, structural lung disease, n (%)	34 (26.2%)	19 (14.6%)	15 (11.5%)	0.33
Hypokalemia, n (%)	11 (8.4%)	5 (3.8%)	6 (4.6%)	> 0.99
Hypomagnesemia, n (%)	5 (3.8%)	4 (3.1%)	1 (0.8%)	0.20
Hypocalcemia, n (%)	16 (12.3%)	8 (6.2%)	8 (6.2%)	> 0.99
Taking QTc-prolonging medications	46 (35.4%)	15 (11.5%)	31 (23.8%)	0.010**
Admission status				
Outpatient, n (%)	8 (6.2%)	0	8 (6.2%)	0.006**
Inpatient, n (%)	122 (93.8%)	63 (48.5%)	59 (45.4%)	

Patients with prolonged QTc were more likely to have diabetes (24.6%) than those with normal QTc (13.1%, p = 0.004). Prolonged QTc patients were more likely to have heart failure (11.5%) than those with normal QTc (3.1%; p = 0.005), and 48.5% with prolonged QTc were inpatients, whereas 45.4% with normal QTc were inpatients (p = 0.006). This can be explained by previous and/or ongoing injury to cardiac muscles or cardiac conduction systems affecting the QTc.

Age, gender, race, and other independent risk factors of QTc prolongation were analyzed but did not significantly affect our results. This may suggest that COVID-19 infection is a possible factor for QTc prolongation. Interestingly, fewer patients with prolonged QTc were taking QTc-prolonging medications than patients in the normal QTc group, supporting the direct effects of COVID-19 on QTc prolongation (normal 23.8% vs. long QTc 11.5%; p = 0.01) independent of QTc medications.

Univariable and multivariable regression analyses were performed, and univariable analysis was conducted for statistically significant variables only. On univariable analysis, the odds of QTc prolongation in patients with diabetes was high (odds ratio (OR) = 2.52; 95% confidence interval (CI) = 1.0 to 5.9; p = 0.033).

The odds of having QTc prolongation were also high in ESRD (OR = 2.53, 95% CI = 0.62 to 10.2), chronic obstructive pulmonary disease and structural lung disease (OR = 1.34, 95% CI = 0.55- 3.24), heart disease (OR = 1.19 95% CI = 050 to 2.84), and electrolyte deficiencies (OR = 1.15, 95% CI = 0.43 to 3.0), but it was not statistically significant. The odds of having prolonged QTc in patients on QTc-prolonging medications were low (OR = 0.29, 95% CI = 0.12 to 0.67; p = 0.004) (Table 2).

**Table 2:** Clinical factors associated with QTc prolongation. HF: heart failure; CAD: coronary artery disease; ESRD: end-stage renal disease

Clinical factors	Odds ratio	95% confidence interval		P-value
Diabetes	2.527	1.079	5.92	0.033
Heart disease (HF, CAD)	1.195	0.502	2.848	0.68
Lung disease (COPD, asthma)	1.343	0.556	3.247	0.51
ESRD	2.538	0.627	10.276	0.19
Electrolyte abnormalities	1.15	0.435	3.039	0.77
Medication prolong QTc	0.294	0.128	0.675	0.004

Our study did not show statistically significant differences in inpatient mortality or outcomes during hospitalization between the two groups. Six (4.6%) participants died from severe cardiac arrhythmia such as VT or Vfib detected on telemonitor. Although this was not statistically significant (long QTc: 0.8%; normal QTc: 3.8%; p = 0.21), it was clinically significant because early recognition of QTc prolongation can lead to the early intervention by providers in preventing serious events (Table 3).

**Table 3:** QTc prolongation effect on hospital course and clinical outcomes. SD: standard deviation

Clinical course/outcome	Total (N = 130)	Long QTc (n = 63)	Normal QTc (n = 67)	P-value
Death, all-cause mortality (n, %)	6 (4.6%)	1 (0.8%)	5 (3.8%)	0.21
Mean length of hospital stay, days (±SD)	6.07 (21.2)	6.44 (4.82)	6.58 (5.91)	0.89

We found no statistically significant differences between the two groups in the length of stay, all-cause mortality, medication, or abnormal electrolytes. Overall, 18% of participants continued to have long QTc after COVID-19. Hospital length of stay was longer for patients with a higher heart rate on admission, especially those with a heart rate above 91 beats per minute (44.6%). The mean length of stay was 7.1 days (range = 1-31 days).

A total of 61 (46.9%) patients showed an increase in QTc, with an average increase of 38.5 ms from baseline (range = 1-178 ms) during SARS-CoV-2 infection. In total, 31 (23%) patients had a long baseline QTc, and 16 (51%) showed an increased QTc duration, with an average increase of 31.2 ms from the baseline, which is a high risk for serious arrhythmia (Table 4).

**Table 4:** Average of QTc prolongation during COVID-19 infection. COVID-19: coronavirus disease 2019

	Total participants	Percentage	Average increase in QTc	Range of QTc change
Total participants with prolonged QTc	61 patients	46.90%	38.5 mesc	1 ms to 178 ms
Total participants with baseline long QTc	31 patients	23%		
Participants with both baseline long QTc and increased QTc during COVID-19 infection	16 patients	51%	31.2 ms	
One month after discharge with persistent QTc prolongation	13 patients	21.5 %	42.7 ms	1 ms to 229 ms

One month after discharge, 21.5% of the participants showed a persistent increase in QTc, with a mean of 42.7 ms from baseline (1-229 ms). Although this may indicate long-term cardiac injury from COVID-19, it requires further investigations.

## Discussion

During the COVID-19 pandemic case surge, more than 20% of our hospitalized COVID-19 patients had long QTc with a strong association with diabetes, ESRD, QTc-prolonging medications, and heart failure. Other risk factors were not statistically significant, such as age, abnormal electrolytes, CAD, and HTN. Our results highlight the direct effect of COVID-19 on QTc prolongation.

SARS-CoV-2 infections are now a major cause of mortality and morbidity worldwide [1]. Since the start of the pandemic, researchers have strived to better understand the disease, appropriate treatment options, and preventative measures to help avoid the virus or its fatal outcomes. During the COVID-19 pandemic, many patients admitted to our hospital had ECGs with prolonged QTc, suggesting a possible correlation between long QTc and COVID-19. Few studies have discussed poor outcomes in COVID-19-related ECG changes [2-5]. A few investigators focused on the correlation between ECG changes, arrhythmia, and QTc prolongation. These studies observed increased tachyarrhythmia and QTc prolongation due to COVID-19 [6-9]. Other studies explored the significant relationship between COVID-19 and biomarkers such as BNP and troponin. These studies tried to examine the relationship between COVID-19 infection and myocardial injury. According to these studies, the results may be due to the possibility of non-ST-elevation myocardial infarction, myocardial cell injury, or cardiomyopathy secondary to COVID-19; moreover, these biomarkers serve as a predictor of poor outcomes but do not explain or specify the relationship to the ECG changes, which triggered our study [11-14]. Additional studies focused on the effect of medications such as remdesivir and hydroxychloroquine on QTc as COVID-19 treatment. These studies showed that both medications increased the risk of QTc prolongation but did not discuss whether COVID-19 was a confounding factor [15,16].

In understanding COVID-19-related cardiac injury, although the pathophysiology during COVID-19 is continuously expanding, one of the most popular theories is that angiotensin-converting enzyme-2 receptors are important for SARS-CoV-2 to enter the host cells. Because these receptors are present in the heart muscle and lungs, they could be the mechanism behind the cardiorespiratory manifestations of COVID-19. However, this hypothesis requires further investigation [20,21]. Theoretically, the mechanisms of cardiac cell injury are hypoxia, myocarditis, myocardial ischemia, or abnormal host immune responses that induce cardiac arrhythmias due to direct viral invasion and inflammation that can result in unstable myocardium and cell membranes and can influence QTc prolongation in COVID-19 [2,22].

The limitations of our study are the presence of confounding factors such as chronic diseases or concurrent use of antipsychotic medications, antibiotics, or electrolyte imbalance that would affect QTc without any effect from COVID-19. Moreover, this study was an observational study.

## Conclusions

We conducted this study to examine the association between COVID-19 and QTc changes. In this study, outcomes and length of hospital stay were unaffected by QTc changes. We encourage scientists and researchers to study this area and the possibility of COVID-19 inducing QTc prolongation. Physicians should consider this possibility when treating cardiac patients with possible or confirmed COVID-19 infection.
